# Anti-influenza A Virus Effects and Mechanisms of Emodin and Its Analogs via Regulating PPAR*α*/*γ*-AMPK-SIRT1 Pathway and Fatty Acid Metabolism

**DOI:** 10.1155/2021/9066938

**Published:** 2021-09-09

**Authors:** Yufei Bei, Boyu Tia, Yuze Li, Yingzhu Guo, Shufei Deng, Rouyu Huang, Huiling Zeng, Rui Li, Ge-Fei Wang, Jianping Dai

**Affiliations:** ^1^Department of Pharmacy, Affiliated Hospital of Nantong University, 20th Xisi Road, 226 001 Nantong, China; ^2^Department of Microbiology and Immunology, Shantou University Medical College, Xinling Road, 22, Shantou, Guangdong 515 041, China

## Abstract

The peroxisome proliferator-activated receptor (PPAR) *α*/*γ*-adenosine 5′-monophosphate- (AMP-) activated protein kinase- (AMPK-) sirtuin-1 (SIRT1) pathway and fatty acid metabolism are reported to be involved in influenza A virus (IAV) replication and IAV-pneumonia. Through a cell-based peroxisome proliferator responsive element- (PPRE-) driven luciferase bioassay, we have investigated 145 examples of traditional Chinese medicines (TCMs). Several TCMs, such as *Polygonum cuspidatum*, *Rheum officinale* Baillon, and *Aloe vera* var. Chinensis (Haw.) Berg., were found to possess high activity. We have further detected the anti-IAV activities of emodin (EMO) and its analogs, a group of common important compounds of these TCMs. The results showed that emodin and its several analogs possess excellent anti-IAV activities. The pharmacological tests showed that emodin significantly activated PPAR*α*/*γ* and AMPK, decreased fatty acid biosynthesis, and increased intracellular ATP levels. Pharmaceutical inhibitors, siRNAs for PPAR*α*/*γ* and AMPK*α*1, and exogenous palmitate impaired the inhibition of emodin. The in vivo test also showed that emodin significantly protected mice from IAV infection and pneumonia. Pharmacological inhibitors for PPAR*α*/*γ* and AMPK signal and exogenous palmitate could partially counteract the effects of emodin in vivo. In conclusion, emodin and its analogs are a group of promising anti-IAV drug precursors, and the pharmacological mechanism of emodin is linked to its ability to regulate the PPAR*α*/*γ*-AMPK pathway and fatty acid metabolism.

## 1. Introduction

Highly pathogenic influenza A virus (IAV) infection or seasonal IAV infection of patients with basal metabolic diseases usually leads to acute lung injury (ALI) and acute respiratory distress syndrome (ARDS). And unfortunately, now, there is no specific medicine available for the treatment of ALI/ARDS. In addition, it is well known that it is difficult to control IAV infection through vaccination due to the rapid antigenic variation through “antigenic drift” and “antigenic shift.” And furthermore, classical anti-IAV drugs, such as M2 channel and neuraminidase inhibitors, are also limited in use by their side effects or the resistant viral strains [1]. In consequence, the development of novel anti-IAV drugs is still an urgent need.

To research the synergistic effects of traditional Chinese medicines (TCMs) and their active compounds, we have engaged in the classification of TCMs according to the different IAV pathogenic mechanisms for many years. In recent years, the peroxisome proliferator-activated receptor (PPAR) *α*/*γ* signaling pathway has been shown to be an antiviral innate immune signal that can protect against lethal IAV attacks in mice [2, 3]. It has been reported that PPAR*α* agonist gemfibrozil can increase the survival of IAV (H2N2)-infected mice from 26% to 52% [2]. PPAR*γ* agonists rosiglitazone, pioglitazone, and 15d-PGJ2 can reduce viral titer and protect mice from lethal IAV infection [3].

PPAR*α*, sirtuin-1 (SIRT1), and adenosine 5′-monophosphate- (AMP-) activated protein kinase (AMPK) can synergistically work together. PPAR*α* can stimulate the expression of SIRT1. SIRT1 can positively regulate AMPK activity [4], and all of PPAR*α*, AMPK, and SIRT1 can inhibit NF-*κ*B signaling and suppress inflammation [5, 6]. Activation of the NF-*κ*B signaling pathway can suppress the SIRT1/peroxisome proliferator-activated receptor-*γ* coactivator-1*α* (PGC-1*α*) pathway and switches on the aerobic glycolysis during acute inflammation [7]. In addition, PPAR*α*/*γ*-AMPK and nuclear factor erythroid 2-related factor 2 (Nrf2)/antioxidant response element (ARE) pathways also can interact mutually, constitute a positive feedback loop, and repress inflammation [8].

In addition, it has been reported that energy metabolic disorder, also named “mitochondrial energy crisis,” is a major risk factor for severe IAV infection [9]. During the late phase of IAV infection, new treatment options have been proposed to target the energy crisis by restoring glucose and long-chain fatty acid oxidation, rather than antiviral treatments with neuraminidase inhibitors [9]. It has been reported that fatty acid metabolism is very important for the replication of many viruses. IAV infection can impair fatty acid oxidation [10] and upregulate fatty acid biosynthesis by increasing the levels of acetyl-CoA carboxylase (ACC) and fatty acid synthetase (FAS), which are two important enzymes for de novo fatty acid biosynthesis; ACC inhibitor 5-tetradecyloxy-2-furoic acid (TOFA) and FAS inhibitor C75 can inhibit IAV replication by >1,000-fold and >10-fold, respectively [11, 12].

PPAR*α*/*γ* pathways play important roles in energy metabolism. PPAR*α* agonists can increase the expression of fatty acid oxidation enzymes, improve mitochondrial membrane potential (*ΔΨ*m), restore the ATP level, and reduce virus production [13]. In the recent study, based on a PPAR response element luciferase reporter (pPPRE-luc), we have screened 145 examples of TCMs and found that several TCMs, such as *Polygonum cuspidatum*, *Rheum officinale* Baillon, and *Aloe vera* var. Chinensis (Haw.) Berg, had high activity. In the following research, we have investigated the anti-IAV effects and mechanisms of emodin (1,3,8-trihydroxy-6-methylanthraquinone) and emodin analogs, all of which are important compounds of these TCMs [14].

## 2. Materials and Methods

### 2.1. Viruses and Cells

All experiments involving viral infection were performed under BSL-3 condition. IAV strains A/PuertoRico/8/34 (PR8, H1N1), A/ShanTou/16/09 (ST169, H1N1), A/ShanTou/1233/06 (ST1233, H1N1), A/ShanTou/602/06 (ST602, H3N2), A/ShanTou/364/05 (ST364, H3N2), A/Quail/HongKong/G1/97 (HKG1, H9N2), A/Chicken/Guangdong/A1/03 (GDA1, H9N2), and A/Chicken/Guangdong/1/05 (GD105, H5N1) were obtained from the Department of Microbiology and Immunology, Shantou University Medical College (Shantou, China). Viral stocks were propagated in Madin-Darby canine kidney cells (MDCK) or 10-day-old embryonated chicken eggs for 72 h. Virus titer was determined by a plaque assay. The cytotoxicity of the test drugs was evaluated by a MTT method, and the time of incubation is 48 h [15].

### 2.2. Plant Extraction and Compounds

TCMs were purchased from Yulin (Guangxi Province, China) and Pulin (Guangdong Province, China) medicine markets and were extracted according to the Chinese Pharmacopoeia (2000). Each specimen was deposited in our lab. The extracts of TCMs were deposited and protected from light in a -20°C refrigerator. Emodin and emodin analogs (Supplementary Figure 1) were purchased from MedChemExpress Co., Ltd (New Jersey, USA). Compound C, MK886, and GW9662 were purchased from Sigma-Aldrich Chemical Co. (St. Louis, USA). All other chemicals were analytical reagent grade.

### 2.3. *In Vitro* Cell Infection and Plaque Assay

In the *in vitro* cell infection assay, the viruses were pretreated with virus growth medium (VGM) containing the test drug for 2 h. VGM is a MEM medium, which contains 1 *μ*g/mL TPCK-trypsin and 3.2% bovine serum albumin. After pretreatment, the viruses were washed with PBS three times and concentrated by ultrafiltration. Before the experiment, MDCK or A549 cells were seeded into six-well plates for 24 h. Then, the pretreated viruses were added and adsorbed for 1 h (multiplicity of infection (MOI) = 0.001) and washed with PBS three times, and the cells were cultivated in a series of mediums containing test drugs for 48 h. After frost-thawing one time, the supernatants were collected and the titers were determined using a plaque assay, or the cells were used in qRCR or western assays.

In the plaque assay, MDCK cells were seeded into six-well plates for 24 h. After washing with PBS three times, the cells were incubated with 0.2 mL of the collected supernatants (virus suspension) at 36°C for 60 min with frequent shaking. After discarding the supernatant and washing with PBS three times, a 0.6% agarose (1 mL) containing 1x VGM overlaid the plates. The plates were incubated at 36°C in a humidified atmosphere of 5% CO_2_ in air. After 36 to 72 h, the plaques were stained with 1% crystal violet solution and counted.

### 2.4. Primary Screening of TCMs and Antiviral Assays

In the primary screening of TCMs, A549 cells (4 × 10^4^) were seeded in 96-well microplates for 24 h, then cotransfected with pPPRE-luc plasmid (Beijing Biolab Technology Co., Ltd., Beijing, China) and pRL-TK plasmid (internal control) using Lipofectamine™ 2000 Transfection Reagent (Invitrogen, Carlsbad, USA). After 8 h, the cells were infected and treated with different drugs (including the positive drug (gemfibrozil) and the extracts of TCMs). After 24 h, the luciferase activity was determined following the instruments of the Luciferase Reporter Assay Kit (BD Biosciences Clontech, CA, USA). The *Z*′-factor, a statistical parameter to quantify the suitability of HTS, was calculated as previously reported [16]. In addition, to determine the antiviral activities of the test drugs (including emodin and its analogs), three methods were performed: plaque inhibition assay, qRT-PCR assay, and MTT method [15].

### 2.5. Assays for Determining the Level of FAS Activity, Free Fatty Acid, and Intracellular ATP

The FAS activity assay kit (BC0550) and free fatty acid quantification assay kit (BC0595) were purchased from Beijing Solarbio Science and Technology Co., Ltd (Beijing, China). The Luminescent ATP Detection Assay Kit was purchased from Abcam (MA, USA). After IAV infection and drug treatment for 48 h, A549 cells were harvested in cold PBS; FAS activity and the level of intracellular free fatty acid and ATP were measured according to the manufacturer's protocols.

### 2.6. Real-Time Quantitative PCR

Total RNA from A549 cells or lung tissues was extracted using the TRIzol reagent kit (Invitrogen, Carlsbad, USA). The mRNA expression levels were determined by real-time PCR using the SYBR Green PCR kit (Qiagen, Hilden, Germany). The results were expressed as 2^-*ΔΔ*Ct^ and normalized to the amount of GAPDH mRNA [17]. The primers are shown in Supplement Table 1.

### 2.7. Western Blot Assay

The western blotting assay was performed as previously reported [18]. Antibodies against *β*-actin, PPAR*α*, PPAR*γ*, malonyl-CoA decarboxylase (MLYCD), sterol-regulatory element binding protein- (SREBP-) 1c (Abcam MA, USA), AMPK*α*, p-AMPK*α*, ACC, p-ACC, fatty acid-binding protein (FABP) 5 (CST, MA, USA), CD36, carnitine palmitoyltransferase (CPT) 1A, CPTII, acyl coenzyme A oxidase (ACOX) 1, and FAS (Santa Cruz Biotechnology, CA, USA) were used. For visualization, the membranes were incubated with HRP-conjugated secondary antibodies (Santa Cruz Biotechnology, CA, USA) and detected by an ECL kit (GE Healthcare Life Sciences, Pittsburgh, PA, USA) according to the manufacturer's instructions. The blots showed were representative of at least three independent experiments. The protein levels were semiquantified by ImageJ software.

### 2.8. RNA Interfering Assay

Human PPAR*α* siRNA (sc-36307), PPAR*γ* siRNA (sc-29455), AMPK*α*1 siRNA (sc-45312), and scrambled siRNA (sc-37007) were purchased from Santa Cruz Biotechnology Inc. (CA, USA) and were used for gene silencing experiments in vitro; the assay was performed according to the manufacturer's instructions.

### 2.9. ELISA Assay

IL-1*β*, IL-6, IL-8, and TNF*α* ELISA kits were purchased from Dakewe (Beijing, China). The IP-10 ELISA kit was purchased from ThermoFisher Scientific (Waltham, MA, USA). The mouse p-AMPK ELISA kit and pACCase ELISA kit were purchased from SenBeiJia Biological Technology Co., Ltd. (Nanjing, China) and Yanjin Biological Technology Co., Ltd. (Shanghai, China), respectively. Cytokines were quantified following the manufacturer's instructions.

### 2.10. *In Vivo* Experiment

Half male and female SPF BALB/c mice (6-8 weeks old) were purchased from Shanghai Slack Laboratory Animal Co., LTD (Shanghai, China). All experimental protocols and euthanasia procedures were approved by the Ethical Animal Experimentation Committee of the University of Shantou University. The mice were housed in a temperature-controlled environment (22–24°C) on a 12 h light/dark cycle (light from 8:30 AM to 8:30 PM) with free access to food and water. After 1 week of adaptive feeding, the median lethal dose was first determined; then, the mice were randomly divided into 8 groups (*n* = 16 in each group). Before the experiment, the mice were anesthetized by intraperitoneal injection of ketamine (100 mg/kg). In the uninfected control (normal group), mice were not infected with IAV (PR8) virus but intranasally shammed with VGM medium and treated with PBS+DMSO (0.5%) by oral gavage. In the negative control (IAV group), positive control (oseltamivir, Ose group), and emodin-treated groups (EMO group), mice were intranasally infected with 50 *μ*L IAV (PR8) virus solution (2.46 × 10^6^ PFU) and orally treated with PBS+DMSO (0.5%), oseltamivir (10 mg/kg/day), and emodin (75 mg/kg/day) from -1 to 5 post infection (p.i.), respectively.

In the inhibitor-treated groups, after IAV infection and emodin treatment, mice were simultaneously treated with the inhibitors of PPAR*α* (MK886, 1 mg/kg/d), PPAR*γ* (GW9662, 2 mg/kg/d), AMPK (Compound C, 20 mg/kg/d), fatty acid *β*-oxidation inhibitor etomoxir (ETO, 30 mg/kg/day), and fatty acid palmitate (PA, 300 mg/kg/day) by intraperitoneal injection, respectively.

Ten mice from each group were observed for morbidity daily and weighed for 14 days. The other 6 mice of each group were put to death by dislocating their cervical vertebras on day 6 p.i. The lung index was assessed by determining the percent of lung wet weight (g) to body weight (g). Right lung tissues were frozen for western blot, qRT-PCR, ELISA, and viral titer assays, and left lung tissues were fixed in 10% neutral buffered formalin (NBF) for pathological analyses. The severity of histological changes was scored according to a semiquantitative scoring method [19].

### 2.11. Statistical Analysis

The statistical significance was assessed using SPSS16.0 software. Data were analyzed by one-way analysis of variance (ANOVA). The survival time was analyzed by Kaplan-Meier analysis with log-rank and Breslow tests. Results are expressed as the mean ± standard deviations (SD). *P* ≤ 0.05 was considered significant.

## 3. Results

### 3.1. Results of Primary Screening and the Anti-IAV Effects of Emodin and Its Analogs *In Vitro*

**Figure 1:**
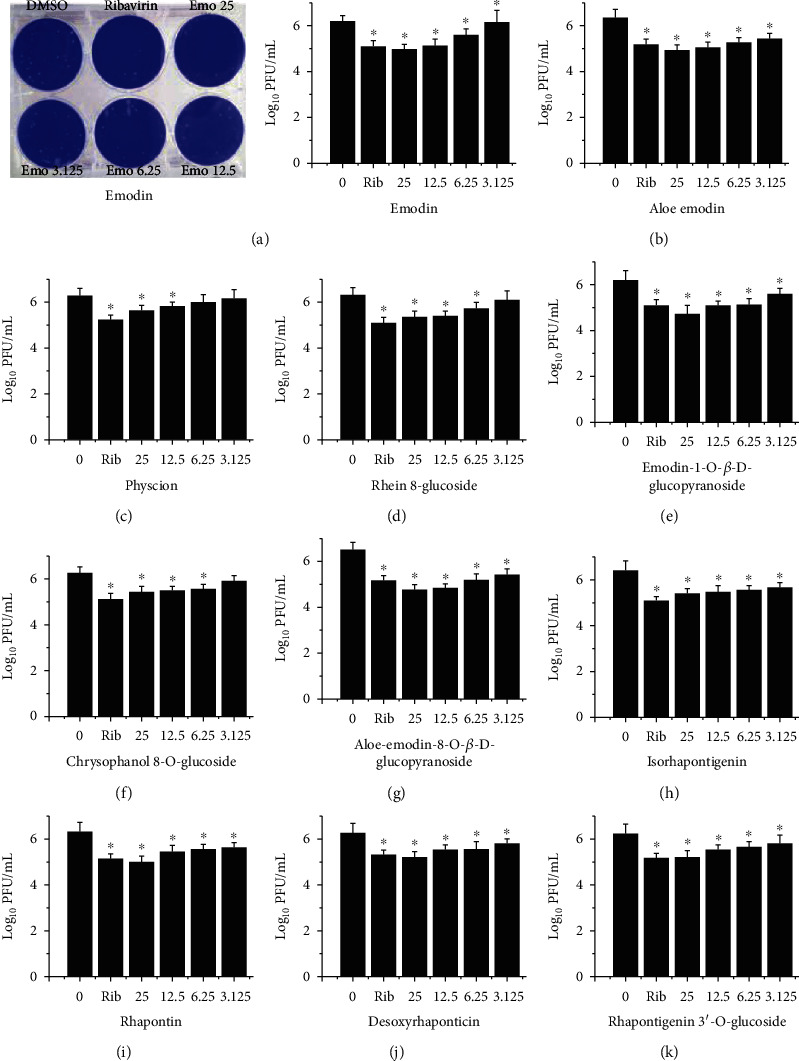
The anti-IAV activity of emodin and its analogs was detected by a plaque inhibition assay. After infection with IAV (PR8, MOI = 0.001), MDCK cells were treated with DMSO (<0.5%), ribavirin (25 *μ*g/mL), and emodin or its analogs (25, 12.5, 6.25, and 3.125 *μ*g/mL), respectively. The incubation time was 48 h. Data shown were the mean ± SD of three independent experiments. ^∗^*P* < 0.05 vs. DMSO-treated group.

In this study, through a cell-based PPRE-driven luciferase bioassay, 145 examples of TCMs were investigated. As shown in Supplement Table 2, several TCMs showed high activity, such as *Polygonum cuspidatum*, *Rheum officinale* Baillon, and *Aloe vera* var. Chinensis (Haw.) Berg. After checking the pharmacology database of traditional Chinese Medicine (https://tcmspw.com/tcmsp.php) and the traditional Chinese medicine integrated database (https://tcm.scbdd.com/home/search_index/) as well as historical literary data [20], we found that emodin was an important compound of these TCMs. Followingly, we further discover the anti-IAV effects of emodin and its analogs, which are a group of anthraquinone compounds, existing in many plants and possessing antibacterial, antiviral, anti-inflammatory, and anticancer effects [21]. Before antiviral tests, we determined the cytotoxicity of emodin and its analogs. The results showed that emodin and its analogs had no significant cytotoxicity on A549 and MDCK cells below the concentration of 25 *μ*g/mL (Supplement Figures 2 and 3). To detect their antiviral activity, we performed a plaque inhibition assay (Figure 1) and a qRT-PCR assay (Supplement Figure 4). The results showed that emodin and its analogs could significantly suppress the replication of IAV (PR8) at concentrations from 12.5 to 25 *μ*g/mL. In addition, several emodin analogs, such as emodin-1-O-*β*-D-glucopyranoside, chrysophanol-8-O-glucoside, aloe-emodin-8-O-*β*-D-glucopyranoside, isorhapontigenin, rhapontin, desoxyrhaponticin, and rhapontigenin 3′-O-glucoside, still significantly reduced the replication of IAV even at the concentration of 3.125 *μ*g/mL, which was better than emodin did.

Furthermore, to detect the broad-spectrum anti-IAV activity of emodin, several different IAV strains have been used, including PR8 (H1N1), ST169 (H1N1), ST1233 (H1N1), HKG1 (H9N2), GDA1 (H9N2), GD105 (H5N1), ST602 (H3N2), and ST364 (H3N2). Through a qRT-PCR assay that detected the level of IAV vRNA and a MTT assay that detected the IAV-induced cell injury, we found that, at the concentrations from 6.25 to 25 *μ*g/mL, emodin could significantly inhibit the replication of several IAV strains and reduced the cell injury (Supplement Figure 5).

### 3.2. PPAR*α*/*γ* and AMPK Pathways Play Important Roles in the Anti-IAV Effect of Emodin

**Figure 2:**
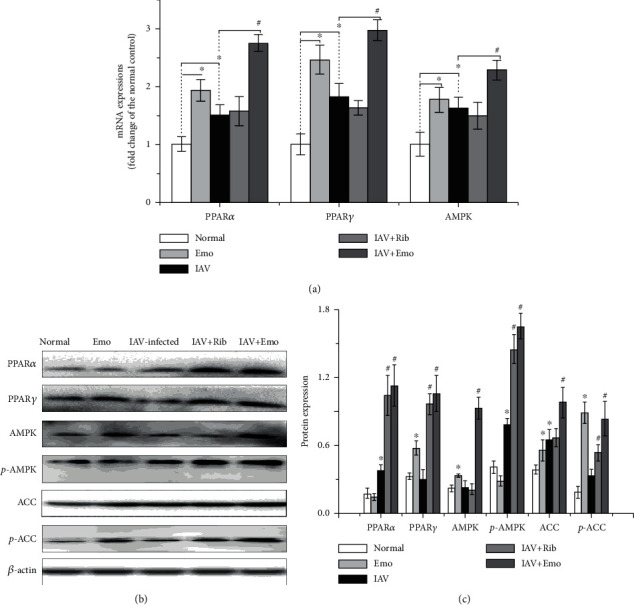
Emodin activated the PPAR*α*/*γ*-AMPK pathway in A549 cells. In the normal and emodin- (EMO-) treated groups, A549 cells were treated with DMSO (0.5% *v*/*v*) and emodin (25 *μ*g/mL), respectively, but not infected with IAV. In the IAV-infected and IAV+Rib- and IAV+EMO-treated groups, A549 cells were infected with IAV (PR8, MOI = 0.001) and simultaneously treated with DMSO (0.5% *v*/*v*), ribavirin (Rib, 25 *μ*g/mL), and emodin (EMO, 25 *μ*g/mL), respectively. After 48 h, the mRNA levels of PPAR*α*, PPAR*γ*, and AMPK were quantified by a qRT-PCR assay (a). The protein levels of PPAR*α*, PPAR*γ*, AMPK, p-AMPK, and p-ACC were quantified by a western blotting assay by using ImageJ software. The results were expressed as the ratio of the target gene to *β*-actin (b, c). All data shown were the mean ± SD of three independent experiments. ^∗^*P* < 0.05 vs. the mock-treated group; ^#^*P* < 0.05 vs. the only IAV-infected group.

Further, we chose emodin as our target compound to investigate the mechanism of action of these compounds. In this study, we first investigated the influences of emodin on the PPAR*α*-AMPK signaling pathway. As shown in Figure 2, IAV infection could significantly increase the mRNA and protein expressions of PPAR*α*/*γ* and AMPK and elevate the phosphorylation of AMPK and ACC in A549 cells. ACC is a major downstream substrate of AMPK, and the phosphorylation level of ACC can represent the serine/threonine protein kinase activity of AMPK. Emodin could further significantly increase the mRNA expression of PPAR*α*/*γ* and AMPK and the phosphorylation of AMPK and ACC after IAV infection.

**Figure 3:**
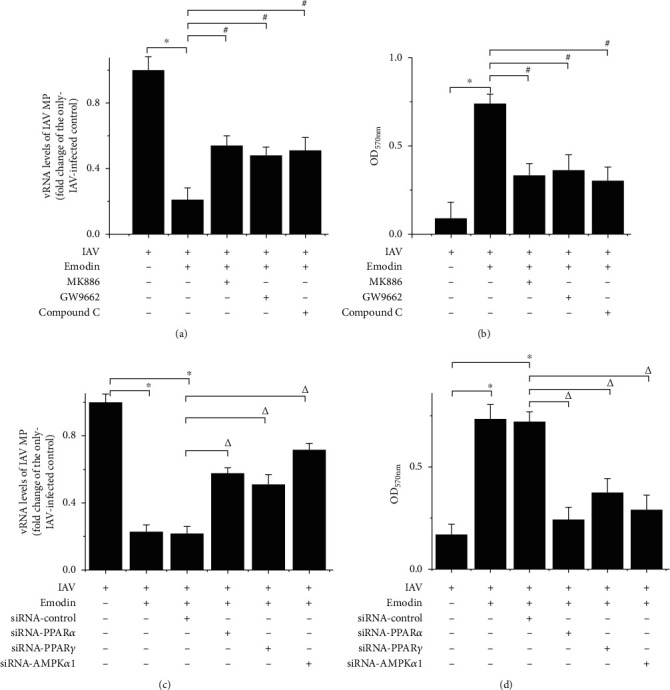
Interference of PPAR*α*/*γ*-AMPK pathway impaired the inhibitory effect of emodin on IAV replication and cell injury. A549 cells were infected with IAV (PR8, MOI = 0.001) and treated with or without emodin (25 *μ*g/mL). (a, b) In the inhibitor-treated assays, A549 cells were simultaneously further treated with the inhibitors of PPAR*α* (MK886, 10 *μ*M), PPAR*γ* (GW9662, 30 *μ*M), and AMPK (Compound C, CC, 10 *μ*M) for 48 h, respectively. The cytotoxicity of these inhibitors was determined (Supplement Figure 6). (c, d) In the siRNA assay, A549 cells were first transfected with the siRNA for PPAR*α*, PPAR*γ*, and AMPK*α*1. After 12 h, the cells were infected with IAV (PR8, MOI = 0.001) and treated with emodin (25 *μ*g/mL) for 48 h. (a, c) The vRNA levels of the IAV matrix protein (MP) gene were quantified by a qRT-PCR assay. (b, d) The counteracting effects of the inhibitors and siRNAs on cell viability were quantified by a MTT method. All data shown were the mean ± SD of three independent experiments each in triplicate. ^∗^*P* < 0.01 vs. the only IAV-infected group; ^#^*P* < 0.05 vs. the IAV+emodin group; *^Δ^P* < 0.05 vs. the IAV+emodin+siRNA control group.

To examine the significance of the PPAR*α*/*γ*-AMPK pathway in emodin-mediated inhibition on IAV replication and IAV-induced injury of cell viability, specific inhibitors of PPAR*α* (MK886), PPAR*γ* (GW9662), and AMPK (Compound C, CC) were used. Through a qRT-PCR assay, we found that emodin-mediated inhibition on IAV replication was significantly counteracted by the treatments of MK886, GW9662, and CC (Figure 3(a)). Through a MTT method, we found that these inhibitors also significantly antagonized the inhibition of emodin on IAV-induced injury of cell viability (Figure 3(b)). Additionally, transfection of siRNAs for PPAR*α*, PPAR*γ*, and AMPK*α*1 also significantly counteracted the inhibition of emodin on IAV replication (Figure 3(c)) and on IAV-induced injury of cell viability (Figure 3(d)). These results indicated that the PPAR*α*/*γ*-AMPK pathway was involved in the inhibition of emodin on IAV replication and IAV-induced cell injury.

**Figure 4:**
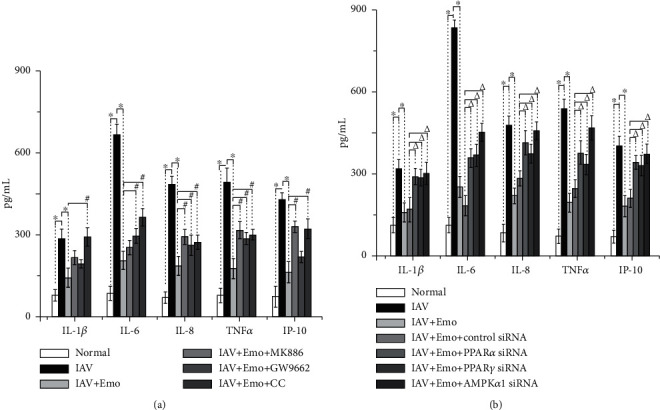
Interference of PPAR*α*/*γ*-AMPK-SIRT1 pathway impaired the inhibition of emodin on IAV-induced production of inflammatory cytokines. A549 cells were infected and treated as mentioned in Figure 2. The levels of cytokines were determined by the ELISA assay. All data shown were the mean ± SD of three independent experiments each in triplicate. ^∗^*P* < 0.01 vs. the only IAV-infected group; ^#^*P* < 0.05 vs. the IAV+emodin group; *^Δ^P* < 0.05 vs. the IAV+emodin+siRNA control group.

In addition, emodin treatment could significantly decrease the IAV-induced increases of IL-1*β*, IL-6, IL-8, TNF*α*, and IP-10. And importantly, treatments with MK886, GW9662, and CC could significantly counteract the inhibition of emodin on IAV-induced inflammatory cytokine production. Moreover, the siRNA for PPAR*α*, PPAR*γ*, and AMPK*α*1 also showed significant antagonistic effects (Figure 4).

### 3.3. Emodin Increased Fatty Acid Oxidation and Decreased Fatty Acid Biosynthesis after IAV Infection

**Figure 5:**
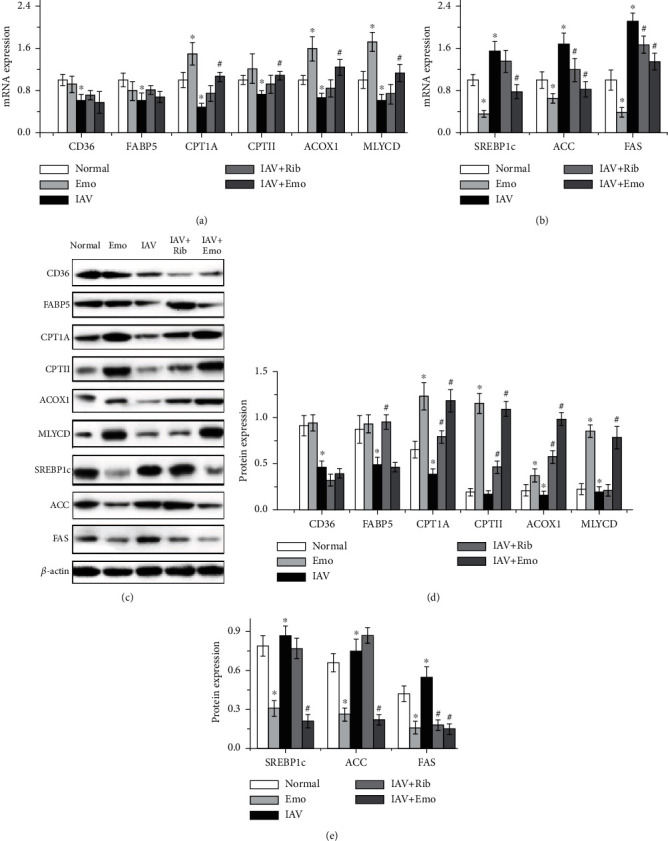
Emodin regulated the gene expression of fatty acid oxidation and fatty acid biosynthesis in A549 cells. The treatments were the same as those in Figure 2. After 48 h, the mRNA and protein expressions were quantified by a qRT-PCR assay (a, b) and by a western blotting assay (c–e). All data shown were the mean ± SD of three independent experiments. ^∗^*P* < 0.05 vs. the normal group; ^#^*P* < 0.05 vs. the only IAV-infected group.

AMPK is a key cellular energy sensor, involved in lipid homeostasis and ATP balance regulation, and most of PPAR agonists exert their physiological effects through activating AMPK. We further determined the influence of emodin on the expressions of genes involved in fatty acid oxidation and biosynthesis. As compared with the IAV-infected control, emodin could further increase the expression of genes involved in fatty acid oxidation (such as carnitine palmitoyl-CoA transferase (CPT) IA, CPTII, acyl-CoA oxidase (ACOX) 1, and MLYCD) and decreased the expression of genes involved in fatty acid biosynthesis (such as SREBP-1c, ACC, and FAS) (Figure 5).

**Figure 6:**
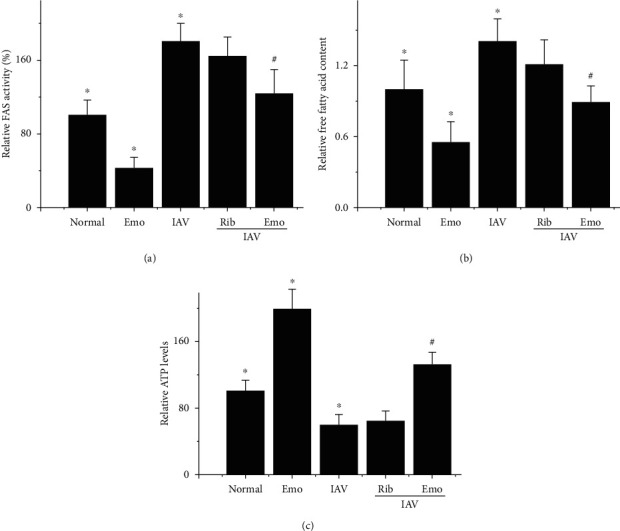
Influence of emodin on FAS activity, free fatty acid, and cellular ATP levels. The treatments were the same as mentioned in Figure 2. After 48 h, relative FAS activity was measured spectrophotometically by monitoring the oxidation of nicotinamide adenine dinucleotide phosphate at 340 nm (a). The levels of free fatty acid and ATP in A549 were also measured by the commercial kits (b, c). Values are presented as the mean ± SD of three independent experiments. ^∗^*P* < 0.05 vs. the normal group; ^#^*P* < 0.05 vs. the only IAV-infected group.

Then, we determined the FAS activity, free fatty acid, and intracellular ATP levels using biochemical methods. The results showed that IAV infection significantly increased the activity of FAS and the level of free fatty acids and decreased the level of intracellular ATP, but emodin significantly reversed these biochemical changes (Figure 6).

**Figure 7:**
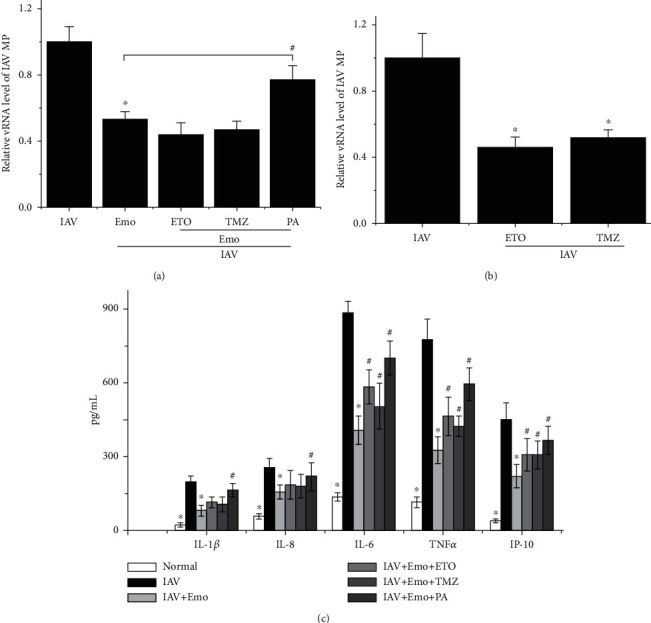
Regulation of *β*-oxidation inhibitors and palmitate on the inhibition of emodin on IAV replication and production of inflammatory cytokines. A549 cells were infected with IAV (PR8, MOI = 0.001) and treated with or without emodin (25 *μ*g/mL). (a) In the antagonism assays, A549 cells were simultaneously further treated with etomoxir (ETO, 100 *μ*M), trimetazidine (TMZ, 8 mM), and palmitate (PA, 100 *μ*M) for 48 h, respectively. (b) To detect the effect of ETO and TMZ on IAV replication, the cells were not treated with emodin. Finally, the vRNA levels of the IAV matrix protein (MP) gene were quantified by a qRT-PCR assay. (c) The levels of cytokines were determined by an ELISA assay. All data shown were the mean ± SD of three independent experiments each in triplicate. ^∗^*P* < 0.01 vs. the only IAV-infected group; ^#^*P* < 0.05 vs. the IAV+emodin group.

Further, we have also determined the significance of fatty acid oxidation and biosynthesis in emodin-mediated anti-IAV effect. We first determined the influence of *β*-oxidation inhibitors (CPT1 inhibitor etomoxir (ETO) and 3-ketoacyl coenzyme A thiolase inhibitor trimetazidine (TMZ)) on the inhibitory effect of emodin. As shown in Figure 7, unexpectedly, ETO and TMZ did not reverse the inhibition of emodin on IAV replication but further reduced the level of IAV vRNA. We further determined the effects of ETO and TMZ on IAV replication without emodin treatment and found that ETO and TMZ themselves could significantly inhibit IAV replication.

Additionally, we further determined the influence of palmitate on IAV infection. Palmitate is the end-product of the de novo fatty acid biosynthesis; the addition of exogenous palmitate can be recognized as the activation of fatty acid biosynthesis. Our results showed that palmitate could impair the inhibitory action of emodin on IAV replication and IAV-induced inflammatory cytokine production (Figure 7).

### 3.4. PPAR*α*/*γ*-AMPK Pathway and Fatty Acid Metabolism Might Play Important Roles in the Inhibitory Effect of Emodin on IAV Infection and Influenzal Pulmonitis *In Vivo*

**Figure 8:**
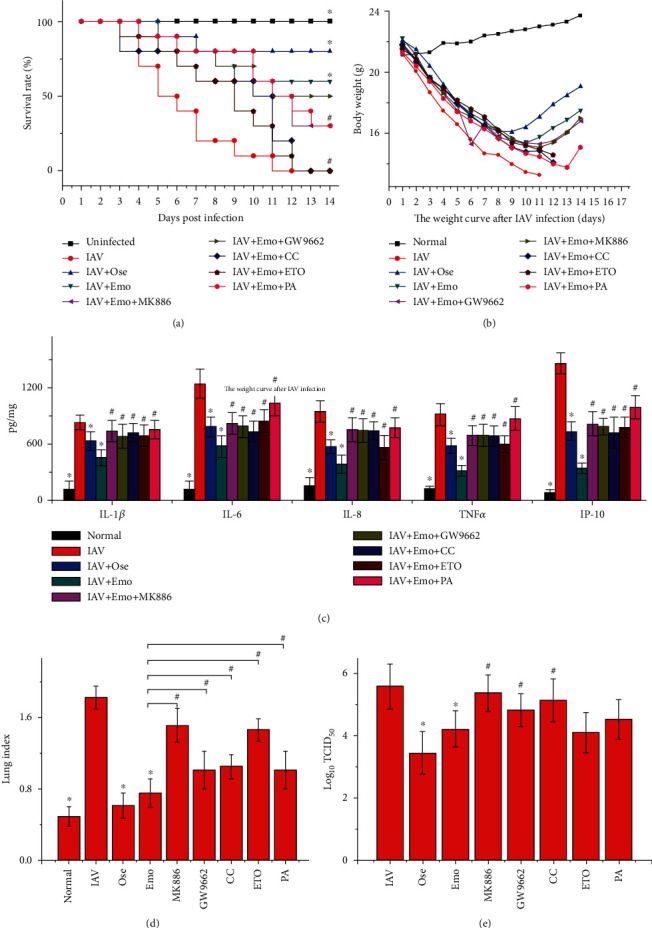
PPAR*α*/*γ*-AMPK pathway and fatty acid metabolism played important roles in emodin-mediated inhibition of IAV infection in mice. In the normal control (normal), mice were not infected with IAV but intranasally shammed with VGM medium. In the only IAV-infected (IAV) and oseltamivir- (Ose-) and emodin- (EMO-) treated groups, mice were infected with 10x MLD50 of IAV (PR8) and treated with PBS+DMSO (<0.5%), oseltamivir (10 mg/kg/d), and emodin (75 mg/kg/d) from -1 to 5 d p.i., respectively. In the antagonism groups, IAV-infected mice were treated with emodin (75 mg/kg/d) by oral gavage and simultaneously treated with the inhibitors of PPAR*α* (MK886, 1 mg/kg/d), PPAR*γ* (GW9662, 2 mg/kg/d), AMPK (CC, 20 mg/kg/d), *β*-oxidation inhibitor (ETO, 30 mg/kg/day), and fatty acid palmitate (PA, 300 mg/kg/day) by intraperitoneal injection from -1 to 5 p.i., respectively. The survival rates were observed for 14 days. The pulmonary cytokine and viral load were determined by ELISA and TCID50 assays, respectively. The lung index was evaluated by determining the percent of lung wet weight (g) to body weight (g) (lung index = lung wet weight (g) ÷ body weight (g) × 100%). ^∗^*P* < 0.05 vs. the IAV-infected group; ^#^*P* < 0.05 vs. the IAV+emodin group.

**Figure 9:**
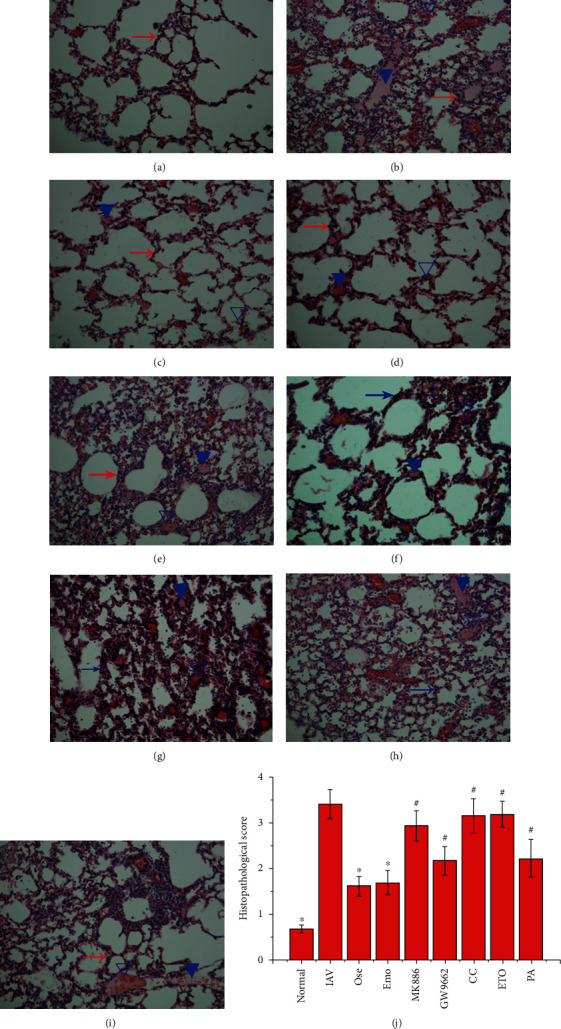
Influence of emodin on the histopathological changes after IAV infection. Mice were treated as mentioned in Figure 8. On day 6 p.i., six mice from each group were sacrificed. The right lungs were used in the haematoxylin and eosin (H&E) staining assay. The magnification was 200x. The evaluation of histopathological scores was carried out in a double-blind trial. Data shown were the mean ± SD. ^∗^*P* < 0.05 vs. the only IAV-infected control; ^#^*P* < 0.05 vs. the IAV+emodin group.

To further determine the importance of the PPAR*α*/*γ*-AMPK pathway and fatty acid metabolism in the anti-IAV activity of emodin, an in vivo test was performed. As shown in Figure 8, comparing with the only IAV-infected control (IAV), emodin treatment (IAV+EMO) could significantly improve the average survival time of infected mice and reduce lung index, lung cytokines, and pulmonary viral load. Furthermore, in the antagonism tests, comparing with the IAV+EMO group, the inhibitors of PPAR*α* (MK886) and AMPK (CC) could significantly counteract the effects of emodin. PPAR*γ* inhibitor GW9662 could partially counteract the effects of emodin. ETO and palmitate could counteract the effects of emodin on average survival time, lung index, and production of lung cytokines, but not significantly on IAV replication. Additionally, emodin could significantly improve IAV-induced histopathological changes, whereas MK886, CC, GW9662, ETO, and PA could partially counteract the inhibition of emodin on IAV-induced histopathological changes (Figure 9).

**Figure 10:**
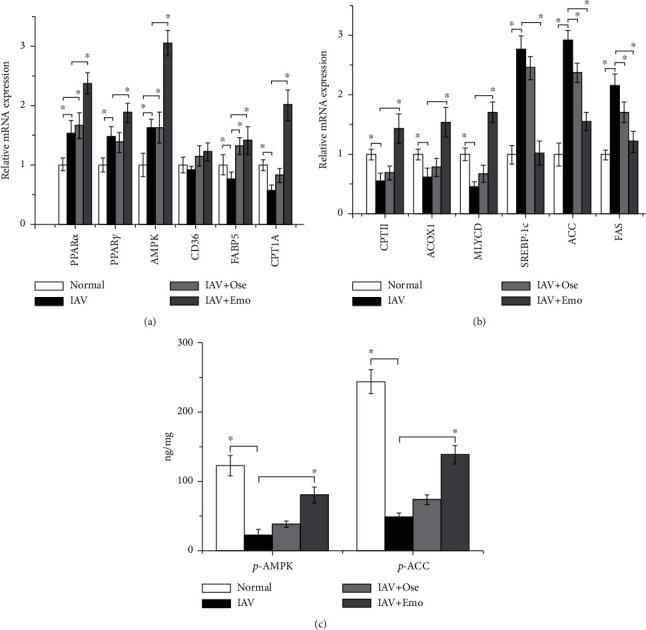
Influence of emodin on PPAR*α*/*γ*-AMPK, fatty acid oxidation, and fatty acid biosynthesis pathways after IAV infection in vivo. Mice were treated as mentioned in Figure 8. On day 6 p.i., lung tissues were collected and homogenated. The mRNA levels of target genes were measured by qRT-PCR (a, b). The phosphorylation levels of AMPK and ACC were determined by an ELISA assay (c). Data shown were the mean ± SD. *n* = 6. ^∗^*P* < 0.05 vs. the only IAV-infected control.

Finally, after IAV infection, emodin also significantly increased the lung mRNA expression of PPAR*α*, PPAR*γ*, AMPK, FABP5, CPT1A, CPTII, ACOX1, and MLYCD and the phosphorylation levels of AMPK and ACC and decreased the mRNA expression of SREBP-1c, ACC, and FAS, but not significantly for CD36 (Figure 10).

## 4. Discussion

In the past more than ten years, we have always engaged in the screening and classification of TCMs and their active compounds by different cell-based luciferase bioassays according to different pathogenic mechanisms of IAV. Recently, we have focused on the PPAR*α*/*γ*-AMPK pathway and performed a cell-based PPRE-driven luciferase bioassay and found that several TCMs have high activity. After further research, we find that emodin is very effective in attenuating IAV replication, which promotes us to further explore the effects of emodin analogs on IAV infection. In this study, we find that several emodin analogs have better anti-IAV activity than emodin does and emodin possesses broad-spectrum anti-IAV effect, both of which suggest that emodin and its analogs are a group of promising anti-IAV drug precursors.

To research the synergistic effect of emodin and its analogs with other TCMs and their active compounds, it is necessary to determine the pharmacological mechanism of emodin and its analogs. In the present study, we researched the pharmacological mechanism of emodin and found that emodin can significantly increase the expressions of PPAR*α*, PPAR*γ*, and AMPK and upregulate the phosphorylation and enzymatic activity of AMPK in A549 cells, no matter IAV infection or not, while treatments with inhibitors or transfections of siRNAs for PPAR*α*, PPAR*γ*, and AMPK*α*1 can significantly impair the inhibitory action of emodin on IAV replication and IAV-induced cell injury, indicating that the PPAR*α*/*γ*-AMPK pathway involves in the inhibition of emodin on IAV replication and pathogenic mechanism. In fact, there are many reports about emodin activating the PPAR*α*/*γ*-AMPK pathway [22].

Moreover, AMPK is a key cellular energy sensor, involved in lipid homeostasis and ATP balance regulation, and most PPAR agonists exert their physiological effects through activating AMPK. The PPAR*α*/*γ*-AMPK pathway also is an important anti-inflammatory pathway. Activation of PPAR*α* can inhibit NF-*κ*B by enhancing the expression of I*κ*B*α* or directly binding to RelA/p65 protein [23]. Activation of PPAR*γ* can inhibit the expressions of TLR4 and NADPH oxidase p47phox [24] and antagonize the inflammatory pathways such as NF-*κ*B, AP1, and STAT in respiratory virus infections [25]. AMPK activation can inhibit oxidative stress and airway inflammation in mice [26]. In our present study, treatments with inhibitors or transfections of siRNAs for PPAR*α*, PPAR*γ*, and AMPK*α*1 can also significantly impair the inhibitory effect of emodin on IAV-induced production of IL-1*β*, IL-6, IL-8, TNF*α*, and IP-10.

It is also well known that the PPAR*α*/*γ* pathway plays an important role in fatty acid metabolism. Bezafibrate, a pan-PPAR agonist, can significantly increase the levels of CD36, ACOX1, CPTI, CPT II, and uncoupling protein-2/3 (UCP-2/3), improve mitochondrial *ΔΨ*m, restore intracellular ATP level, and further improve IAV-associated encephalopathy [27]. PPAR*α* agonists can significantly increase the mRNA expression of fatty acid oxidation enzymes, such as fatty acid transport protein (FATP), FABP, CPTI, CPTII, medium-chain acyl-CoA dehydrogenase (MCAD), long-chain acyl-CoA dehydrogenase deficiency (LCAD), ACOX, peroxisomal ketoacyl-CoA thiolase (KetoACoA), and MLYCD, further improve *ΔΨ*m, restore ATP level, and reduce virus production [13]. PPAR*γ* agonist rosiglitazone also can stimulate the phosphorylation of AMPK*α* and ACC and increase fatty acid oxidation in muscle [28].

Fatty acid oxidation is very important for cell proliferation and functional homeostasis, because the carbons of fatty acid, which substantially replenish the Krebs cycle, are incorporated into aspartate (a nucleotide precursor) and uridine monophosphate (a precursor of pyrimidine nucleoside triphosphates) and finally incorporated into DNA. Reduction of fatty acid oxidation will deplete cell stores of deoxyribonucleoside triphosphates (NTPs) and further impair the de novo nucleotide synthesis for DNA replication [29]. In mitochondrial fatty acid oxidation (*β*-oxidation), CPT1 is the key rate-limiting enzyme that mediates the transportation of fatty acids across the mitochondrial membrane into the matrix [30]. CPT1A overexpression increases long-chain fatty acid oxidation; CPT1A inhibitor etomoxir significantly decreased intracellular ATP levels [31]. Peroxisomes are indispensable for *α*-oxidation of branch chain fatty acids and *β*-oxidation of very long-chain fatty acids (>22 carbons; VLCFA). VLCFA are first oxidized by peroxisomal *β*-oxidation rate-limiting enzyme ACOX1 [32].

IAV infection impairs *β*-oxidation. IAV infection significantly decreases CD36 expression [10]. IAV infection inhibits LCAD and reduces lung function by inhibiting pulmonary surfactant [33]. LCAD^−/−^ mice demonstrated high mortality following IAV infection [34]. Very long-chain acyl-CoA dehydrogenase (VLCAD) deficiency increases brain vascular permeability, enhances the invasion of nonneurotropic IAV into the brain, and causes brain edema [35].

As we expected, emodin could significantly increase the expression of fatty acid oxidation genes, such as CPT1A, CPTII, ACOX1, and MLYCD, and significantly increase the ATP level in A549 cells, no matter IAV-infected or not. But in the antagonism assay, our results have showed that CPT1 inhibitor ETO and 3-ketoacyl coenzyme A thiolase inhibitor TMZ cannot reverse the inhibition of emodin on IAV replication but further reduces IAV replication. A further assay has showed that ETO and TMZ per se can significantly inhibit IAV replication. These results might suggest that *β*-oxidation might also be needed for IAV replication, which we speculate might be due to the fact that fatty acid oxidation is important for the de novo nucleotide synthesis of DNA and is a major source of energy for both cell survival and viral replication. This speculation needs further study to prove.

The *de novo* fatty acid biosynthesis plays an important role in viral replication. The de novo fatty acid biosynthesis is required for the formation of viral envelopment or lipid modification of viral proteins in the replication of many viruses [36]. ACC is the first rate-limiting enzyme in the de novo fatty acid biosynthesis. FAS is the important enzyme that catalyzes the reaction of malonyl-CoA with acetyl-CoA, ultimately generating the 16-carbon fatty acid palmitate. Palmitate is further utilized for the synthesis of more complex glycerophospholipids, sphingolipids, and cholesterol. IAV infection can induce de novo fatty acid biosynthesis and cholesterol synthesis [11]. ACC inhibitor TOFA and FAS inhibitor C75 significantly inhibit IAV replication [12].

The de novo fatty acid biosynthesis is also regulated by AMPK; AMPK is a direct upstream kinase that suppresses the cleavage and nuclear translocation of sterol-regulatory element binding protein (SREBP) 1. SREBP is a key lipogenic transcription factor that regulates the de novo fatty acid biosynthesis by activating genes involved in fatty acid synthesis (such as ACC and FAS) and triglyceride synthesis (such as stearoyl CoA desaturase 1, SCD1) [37]. Activation of AMPK can inhibit the replication of coxsackievirus B3 by inhibiting fatty acid biosynthesis and cellular lipid accumulation. This restriction can be bypassed by treatment with the exogenous palmitate and siRNA AMPK [38].

In the present experiment, emodin can significantly decrease the expression of genes involved in fatty acid biosynthesis (such as SREBP-1c, ACC, and FAS), inhibit FAS activity, and reduce the level of free fatty acid in A549 cells. Exogenous palmitate can significantly impair the inhibitory action of emodin on IAV replication and the production of inflammatory cytokines. In addition, there are some reports about emodin inhibiting fatty acid biosynthesis [39].

Finally, we have performed an in vivo test and have found that emodin treatment can significantly improve the average survival time and reduce lung index, lung cytokines, pulmonary viral load, and histopathological changes, while the inhibitors MK886, GW9662, CC, and ETO as well as palmitate can partially counteract the inhibitory effect of emodin. Emodin also can significantly increase the lung mRNA expression of PPAR*α*, PPAR*γ*, AMPK, FABP5, CPT1A, CPTII, ACOX1, and MLYCD; decrease the mRNA expression of SREBP-1c, ACC, and FAS; and increase the phosphorylation levels of AMPK and ACC. Similar research has reported that emodin significantly ameliorates LPS-induced ALI/ARDS in mice by suppressing LPS-induced downregulation of PPAR*γ* and upregulation of NF-*κ*B p65 [40].

## 5. Conclusion

In the present study, we have performed a cell-based PPRE-driven luciferase bioassay and found that several emodin analogs possess better anti-IAV activity than emodin does, and emodin possesses broad-spectrum anti-IAV activity. These findings suggest that emodin and its analogs are a group of promising anti-IAV drug precursors. The pharmacological mechanism of emodin is related to its ability to regulate the PPAR*α*/*γ*-AMPK pathway and fatty acid metabolism.

## Data Availability

All data used during the study are available in the article and can be solicited from the corresponding authors.

## Supplementary Materials

Supplementary Materials Supplement Table 1: the sequences of primers used in the qRT-PCR assay. Supplement Table 2: results of drug screening based on PPRE luciferase reporter. Supplement Figure 1: emodin analogs. Supplement Figure 2: the cytotoxicity of emodin and its analogs on A549 cells was determined by a MTT assay. Data were the mean ± SD of three independent experiments each performed in triplicate. ^∗^*P* < 0.05 vs. the 0 *μ*g/mL control. Supplement Figure 3: the cytotoxicity of emodin and its analogs on MDCK cells was determined by a MTT assay. Data were the mean ± SD of three independent experiments each performed in triplicate. ^∗^*P* < 0.05 vs. the 0 *μ*g/mL control. Supplement Figure 4: the anti-IAV activity of emodin and its analogs was detected by a qRT-PCR assay. After infection with IAV (PR8, MOI = 0.001), A549 cells were treated with DMSO (<0.5%), ribavirin (25 *μ*g/mL), and emodin or its analogs (25, 12.5, 6.25, and 3.125 *μ*g/mL), respectively. MOI = 0.001. The incubation time was 48 h. All data shown were the mean ± SD of three independent experiments each performed in triplicate. ^∗^*P* < 0.05 vs. 0 *μ*g/mL group. Supplement Figure 5: the broad-spectrum antiviral activity of emodin in vitro. The broad-spectrum anti-IAV activity of emodin was detected using eight IAV strains, including PR8 (H1N1), ST169 (H1N1), ST1233 (H1N1), HKG1 (H9N2), GDA1 (H9N2), GD105 (H5N1), ST602 (H3N2), and ST364 (H3N2), by a qRT-PCR assay and a MTT assay. MOI = 0.001. The incubation time was 48 h. All data shown were the mean ± SD of three independent experiments each performed in triplicate. ^∗^*P* < 0.05 vs. 0 *μ*g/mL group. Supplement Figure 6: the cytotoxicity of these inhibitors was determined at 48 h by a MTT assay. Data were the mean ± SD of three independent experiments each performed in triplicate. ^∗^*P* < 0.05*vs.* the 0 *μ*g/mL control.
