# Supplementary material for: Anti-influenza A Virus Effects and Mechanisms of Emodin and Its Analogs via Regulating PPARα/γ-AMPK-SIRT1 Pathway and Fatty Acid Metabolism
Source: Biomed Res Int. 2021 Sep 9;2021:9066938. doi: 10.1155/2021/9066938 (PMC8445710; doi:10.1155/2021/9066938)
Supplement: Supplementary Materials — Supplement Table 1: the sequences of primers used in the qRT-PCR assay. Supplement Table 2: results of drug screening based on PPRE luciferase reporter. Supplement Figure 1: emodin analogs. Supplement Figure 2: the cytotoxicity of emodin and its analogs on A549 cells was determined by a MTT assay. Data were the mean ± SD of three independent experiments each performed in triplicate. ∗P < 0.05 vs. the 0 μg/mL control. Supplement Figure 3: the cytotoxicity of emodin and its analogs on MDCK cells was determined by a MTT assay. Data were the mean ± SD of three independent experiments each performed in triplicate. ∗P < 0.05 vs. the 0 μg/mL control. Supplement Figure 4: the anti-IAV activity of emodin and its analogs was detected by a qRT-PCR assay. After infection with IAV (PR8, MOI = 0.001), A549 cells were treated with DMSO (<0.5%), ribavirin (25 μg/mL), and emodin or its analogs (25, 12.5, 6.25, and 3.125 μg/mL), respectively. MOI = 0.001. The incubation time was 48 h. All data shown were the mean ± SD of three independent experiments each performed in triplicate. ∗P < 0.05 vs. 0 μg/mL group. Supplement Figure 5: the broad-spectrum antiviral activity of emodin in vitro. The broad-spectrum anti-IAV activity of emodin was detected using eight IAV strains, including PR8 (H1N1), ST169 (H1N1), ST1233 (H1N1), HKG1 (H9N2), GDA1 (H9N2), GD105 (H5N1), ST602 (H3N2), and ST364 (H3N2), by a qRT-PCR assay and a MTT assay. MOI = 0.001. The incubation time was 48 h. All data shown were the mean ± SD of three independent experiments each performed in triplicate. ∗P < 0.05 vs. 0 μg/mL group. Supplement Figure 6: the cytotoxicity of these inhibitors was determined at 48 h by a MTT assay. Data were the mean ± SD of three independent experiments each performed in triplicate. ∗P < 0.05vs. the 0 μg/mL control. [file 9066938.f1.doc]

**Supplement** **Table 1 The sequences of primers used in the qRT-PCR assay.**

| Gene | Forward (5′→ 3′) | Reverse(5′→ 3′) |
| --- | --- | --- |
| hGAPDH | GGTCACCAGGGCTGCTTTTA | GGATCTCGCTCCTGGAAGATG |
| hPPARα | GCTTTGGCTTTACGGAATACCA | TTCGATGTTCAATGCTCCACTG |
| hPPARγ | CCTATTGACCCAGAAAGCGATT | CATTACGGAGAGATCCACGGA |
| hAMPKα1 | ACAGGCATATGGTGGTCCATAGAGA | TTGGTGAGCCACAACTTGTTC |
| hFAT/CD36 | CGCTGAGGACAACACAGTCT | GTTGTCAGCCTCTGTTCCAA |
| hFABP5 | TGAAGGAGCTAGGAGTGGGAA | TGCACCATCTGTAAAGTTGCAG |
| hCPT1a | TGGCGTCTGAGAAGCATCAGCATA | ACACCACGTAAAGGCAGAAGAGGT |
| hCPT II | ACTTGAACCCTGCAAAAAGTGACAC | CATCAGTCAGCTCGAAGTTGAGTTT |
| hACOX1 | GGAACTCACCTTCGAGGCTTG | TTCCCCTTAGTGATGAGCTGG |
| hMLYCD | ACGTCCGGGAAATGAATGGG | GTAACCCGTTCTAGGTTCAGGA |
| hSREBP1 | GCGCCTTGACAGGTGAAGTC | GCCAGGGAAGTCACTGTCTTG |
| hACC | AATAGCGTCTCTAACTTCCTTCAC | CCGTCACTCAGCCGATGTA |
| hFAS | GGACATGGCTTAGAAGTGGAA | TTGGTGTTGCTGGTGAGTG |
| mGAPDH | CAAGGTCATCCATGACAACTTTG | GGCCATCCACAGTCTGG |
| mPPARα | CACCTTCTACGCTCCCGACCCATC | GGAACCAAGCCCCTCCATCCACTG |
| mPPARγ | CAGCAGGTTGTCTTGGATGTC | AGCCCTTTGGTGACTTTATGG |
| mAMPK | GTCAAAGCCGACCCAATGATA | CGTACACGCAAATAATAGGGGTT |
| mFAT/CD36 | AGATGACGTGGCAAAGAACAG | CCTTGGCTAGATAACGAACTCTG |
| mFABP5 | AAAGAGCTAGGAGTAGGACTGG | TGTTGCCATCACACGTAATGA |
| mCPT1a | GATGTTCTTCGTCTGGCTTGA | CTTATCGTGGTGGTGGGTGT |
| mCPTII | CAGCACAGCATCGTACCCA | TCCCAATGCCGTTCTCAAAAT |
| mACOX1 | CCGCCACCTTCAATCCAGAG | CAAGTTCTCGATTTCTCGACGG |
| mMLYCD | GCACGTCCGGGAAATGAAC | GCCTCACACTCGCTGATCTT |
| mSREBP1c | GCCCACAATGCCATTGAGA | GCAAGACAGCAGATTTATTCAGCTT |
| mACC | AATGAACGTGCAATCCGATTTG | ACTCCACATTTGCGTAATTGTTG |
| mFAS | GGTCGTTTCTCCATTAAATTCTCA T | CTAGAAACTTTCCCAGAAATGTTC C |
| IAV M | AGATGAGTCTTCTAACCGAGGTCG | TGCAAAAACATCTTCAAGTCTCTG |

**Supplement Table 2. Results of drug screening based on PPRE luciferase reporter**

| Scientific name | Ratio | Scientific name | Ratio |
| --- | --- | --- | --- |
| No-transfected | 0.514±0.671* | Blank group | 1.00±0.000 |
| Negative group | 1.983±0.319^#^ | Gemfibrozil | 3.255±0.509* |
| *Leonurus Artemisia* (Laur.) S.Y.Hu F | 2.243±0.414 | *Coptis chinensis* Franch. | 2.774±0.296* |
| *Achyranthes bidentata* Bl*.* | 2.317±0.306 | *Kochia scoparia* L. | 1.881±0.286 |
| *Polygonatum sibiricumRed.* | 1.837±0.256 | *Fallopia multiflora* (Thunb.) Harald | 2.023±0.303 |
| *Gardenia jasminoides* Ellis | 2.559±0.276 | *Ophiopogon japonicus* (Linn. f.) K.G. | 1.503±0.127* |
| *Rheum officinale* Baillon | 2.901±0.227* | *Scutellaria baicalensis* Georgi. | 1.487±0.119* |
| *Rhus chinensis* Mill. | 2.487±0.347 | *Bambusa tuldoides* Munro. | 1.901±0.204 |
| *Verbena officinalis* L. | 2.662±0.374 | *Curcuma rcenyujin* Y.H. Chenet C.L. | 2.430±0.338 |
| *Pulsatilla chinensis* (Bge.) Reg. | 1.995±0.209 | *Aloe vera* var. chinensis (Haw.) Berg | 3.013±0.295* |
| *Ilex purpurea* Hassk. | 2.896±0.217* | *Gentiana scabra* Bunge. | 1.793±0.216 |
| *Zingiber officinale* Rosc | 2.223±0.279 | *Peucedanum praeruptorum* Dunn. | 1.767±0.248 |
| *Cimicifuga foetida* L. | 2.203±0.267 | *Lithospermum erythrorhizon* Sieb. et Zucc. | 1.884±0.262 |
| *Caesalpinia sappan* L. | 2.433±0.295 | *Artemisia capillaris* Thunb | 2.338±0.327 |
| *Piper longum* L. | 1.777±0.216 | *Mahonia fortunei* (Lindl.)Fedde | 1.873±0.289 |
| *Fritillaria thunbergii* | 1.946±0.221 | *Agrimonia pilosa* Ledeb. | 1.433±0.199* |
| *Senecio scandens* Buch.-Ham. ex D. Don | 1.953±0.198 | *Hedyotis diffusa* Willd. | 2.792±0.189* |
| *Pueraria lobata* (Willd.) Ohwi. | 2.452±0.273 | *Magnolia denudata* Desr. | 2.053±0.217 |
| *Semiaquilegia adoxoides* (DC.)Makino | 1.609±0.218 | *Eucommia ulmoides* Oliv. | 2.188±0.222 |
| *Belamcanda chinensis* (L.) R. | 2.587±0.338 | *Polygonum cuspidatum* Sieb. et Zucc. | 2.774±0.204* |
| *Atractylodes lancea* ( Thunb.) DC. | 2.111±0.227 | *Dioscorea nipponica* Makino | 2.376±0.279 |
| *Atractylodes macrocephala* | 2.333±0.298 | *Xanthium sibiricun* Patr | 2.319±0.276 |
| *Angelica sinensis* (Oliv.) Diels | 2.911±0.247* | *Commelina communis* L. | 1.754±0.289 |
| *Artemisia carvifolia* | 1.699±0.258 | *Ampelopsis Radix* | 2.586±0.264 |
| *Cirsium japonicum* Fisch. ex DC. | 2.443±0.224 | *Euchresta japonica* L. | 2.268±0.216 |
| *Houttuynia cordata* Thunb. | 2.348±0.276 | *Pyrrosia shearerii* (Bak.) Ching | 2.442±0.264 |
| *Eriobotrya japonica* (Thunb.) Lindl | 2.509±0.189 | *Rubia cordifolia* L. | 2.437±0.208 |
| *Ardisia japonica* (Thunb) Blume | 1.974±0.245 | *Cibotium barometz* | 2.421±0.301 |
| *Evodia lepta* (Spreng.) Merr. | 2.3424±0.199 | *Tadehagi triquetrum* (L.) Ohashi | 1.481±0.119* |
| *Emilia sonchifolia* (L.) DC | 2.439±0.289 | *Forsythia suspensa* (Thunb.) Vahl | 2.119±0.268 |
| *Dryopteris setosa* (Thunb.) Akasawa | 3.175±0.393* | *Lantana camara* L. | 2.462±0.285 |
| *Patrinia scabiosaefolia* Fisch. | 2.259±0.267 | *Siegesbeckia orientalis* L. | 2.339±0.248 |
| *Ginkgo biloba* L. | 1.695±0.299 | *Eriosema chinense* Vogel | 1.516±0.124* |
| *Citrus aurantium* L. | 2.248±0.325 | *Ligusticum sinense* Oliv. | 2.974±0.149* |
| *Phragmites communis* Trin. | 1.632±0.214 | *Ailanthus altissima* (Mill.) Swingle | 2.888±0.215* |
| *Cremastra appendiculata* (D. Don) Makino | 1.767±0.225 | *Fraxinus rhynchophylla* Hance. | 2.065±0.221 |
| *Ligustrum lucidum* Ait. | 2.439±0.258 | *Ziziphus jujuba* Mill. | 3.772±0.379* |
| *Equisetum hiemale* L. | 1.985±0.274 | *Rohdea japonica* Roth. | 1.994±0.304 |
| *Draba nemorosa* L. | 1.678±0.218 | *Andrographis paniculata* (Burm. F.) Nees. | 1.736±0.291 |
| *Lycium chinense* Mill. | 2.49±0.331 | *Scutellaria barbata* D. Don. | 2.833±0.205* |
| *Vitex trifolia* L. | 2.467±0.348 | *Pinellia ternata* | 2.642±0.366 |
| *Citrus reticulate* Blanco. | 2.36±0.271 | *Imperata cylindrica* Beauv. | 3.284±0.375* |
| *Scrophulariae Radix* | 2.534±0.276 | *Lophatherum gracile* | 2.379±0.301 |
| *Centella asiatica* (L.) Urban | 2.59±0.391 | *Brucea javanica* L. | 1.894±0.264 |
| *Gentiana macrophylla* Pall. | 1.866±0.264 | *Pseudolarix kamepferi* Gord | 1.882±0.296 |
| *Aristolochia debilis* Seib.et Zucc. | 2.946±0.257* | *Panax ginseng* C. A. Mey | 3.328±0.346* |
| *Schisandra chinensis* (Turcz.) Baill. | 2.492±0.428 | *Stemona sessilifolia* (Miq.) Miq | 1.825±0.218 |
| *Alangium chinense* (Lour.) *Harms* | 2.393±0.275 | *Sargassum pallidum* (Turn.) C. Ag. | 1.689±0.322 |
| *Plantago depressa* Willd. | 1.994±0.286 | *Buddleja lindleyana*Fort. | 2.341±0.294 |
| *Erodium stephanianum* Willd. | 1.811±0.254 | *Ephedra sinica* Stapf | 3.217±0.384* |
| *Paeonia veitchii* Lynch | 2.213±0.263 | *Hypericum japonicum* Thunb. | 1.919±0.295 |
| *Acanthopanax senticosus* (Rupr. Maxim.) Harms. | 1.849±0.263 | *Centipeda minima* (L.) A. Br. Et Ascher. | 2.453±0.277 |
| *Alsophila spinulosa* (Wall.ex Hook.) R.M.Tryon | 2.163±0.264 | *Euphorbia hirta .* L. | 2.157±0.261 |
| *Pinus tabulaefomis* Carr. | 2.667±0.312 | *Celosia argentea* L. | 2.195±0.285 |
| *Zanthorμlum nitidum* (Roxb.) DC. | 2.338±0.357 | *Hibiscus mutabilis* L | 2.336±0.379 |
| *Saposhnikovia divaricata* (Turcz.) Schischk. | 2.438±0.417 | Buffalo Horn | 1.699±0.394 |
| *Catsia tora* L. | 2.993±0.211* | *Isatis indigotica* Fort． | 2.562±0.395 |
| *Cinnamomum cassia* Presl | 2.449±0.357 | *Lobelia chinensis* Lour. | 2.255±0.354 |
| *Melia toosendan* Sieb. et Zucc. | 1.793±0.305 | *Taxillus sutchuenensis* (Lecomte) Danser | 1.846±0.257 |
| *Phellodendron chinense* Schneid | 2.334±0.362 | *Prunella vulgaris* L. | 2.463±0.335 |
| *Lysimachia christinae* Hance | 2.386±0.304 | *Morus alba* L. | 2.539±0.275 |
| *Ganoderma Lucidum* (Leyss. ex Fr.) Karst. | 3.055±0.291* | *Hypericum ascyron* L*.* | 1.695±0.326 |
| *Galium aparine* L. | 2.439±0.365 | silkworm shit | 1.637±0.285 |
| *Spatholobus suberectus* Dunn | 2.743±0.207* | *Terminalia chebula* Retz. | 1.486±0.118* |
| *Chaenomeles sinensis* (Thouin) Koehne | 2.437±0.353 | *Elaeagnus pungens* | 1.643±0.269 |
| *Lemna minor* | 2.663±0.327 | *Cortex dictamni* | 1.664±0.275 |
| *Serissa serissoides* (DC.) Druce | 1.776±0.238 | *Momordica cochinchinensis* (Lour.) Spreng. | 2.314±0.358 |
| Cicada Slough | 1.813±0.352 | *Paeonia suffruticosa* Andr | 2.537±0.348 |
| *Stemmacantha uniflor*a (L.) Dittrich | 2.259±0.365 | *Lonicera japonica* | 2.517±0.278 |
| *Epimedium brevicornu* Maxim. | 1.457±0.264* | Pseudostellaria heterophylla Miq. | 3.003±0.216* |
| *Euphorbia humifusa* Willd. | 3.366±0.302* | *Herba Artemisiae* Anomalae | 2.773±0.341 |
| *Rhizoma Homalomenae* | 2.479±0.367 | Curcuma zedoaria (Christm.) Rosc. | 1.693±0.389 |
| *Buddleja officinalis* Maxim. | 2.519±0.381 | Codonopsis pilosula (Franch.) Nannf. | 2.284±0.374 |
| *Punica granatum* L. | 3.270±0.348* | *Aster tataricus* L. f. | 3.483±0.261* |
| *Solidago canadensis* L. | 2.113±0.316 |  |  |

In the no-transfected group, A549 cells were not transfected with pPPRE-luc and pRL-TK plasmids and not infected with IAV. In the blank group, A549 cells were co-transfected with pPPRE-luc and pRL-TK plasmids (50:1), but not infected with IAV. In the negative group, A549 cells were co-transfected with pPPRE-luc and pRL-TK plasmids, after 8 h, the cells were infected with IAV, but not treated with any drugs. In the gemfibrozil treated group, A549 cells were transfected with pPPRE-luc and pRL-TK plasmids, infected with IAV, and treated with gemfibrozil. In the drug-treated groups, A549 cells were co-transfected with pPPRE-luc and pRL-TK plasmids, after 8 h, the cells were infected with IAV and treated with the test drugs. As calculated the results, the luciferase activity in each well was first divided by its internal reference, calculated the mean of 3 duplications, then the mean of each group was divided by the mean of the blank control. n = 3, **P* < 0.05 *vs.* the negative group,

^#^*P* < 0.05 *vs.* the blank group.


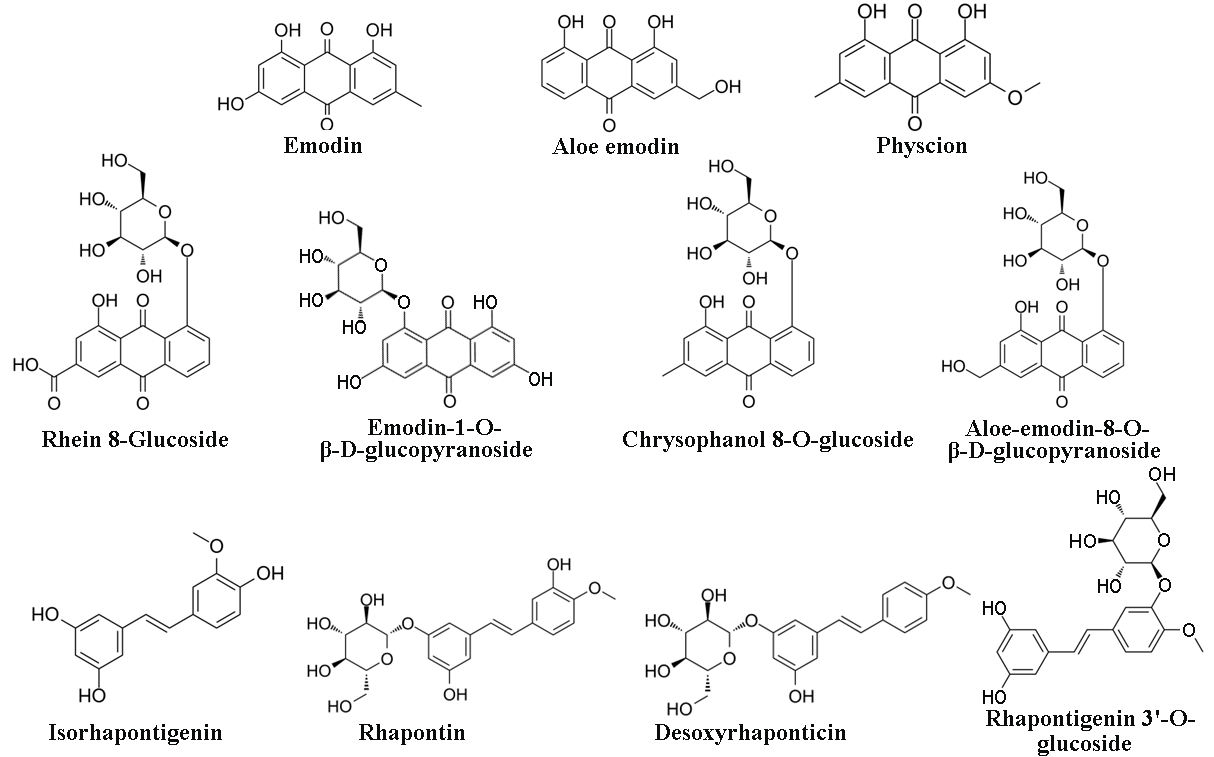


**Supplement figure 1.** Emodin analogs


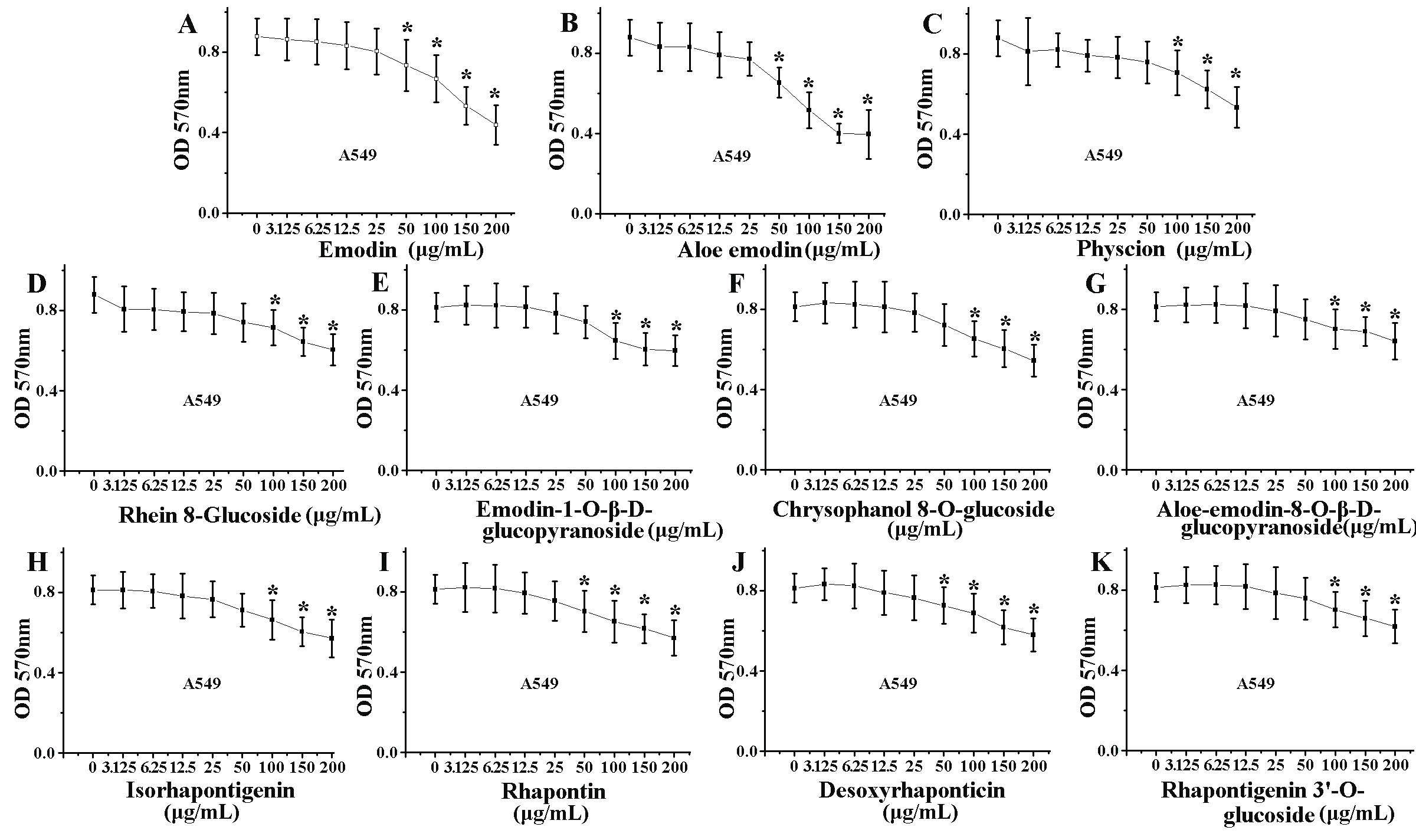


**Supplement Figure 2.** The cytotoxicity of emodin and its analogs on A549 cells was determined by a MTT assay. Data were the mean ± SD of three independent experiments each performed in triplicate. * *P* < 0.05 *vs.* the 0 µg/mL control.


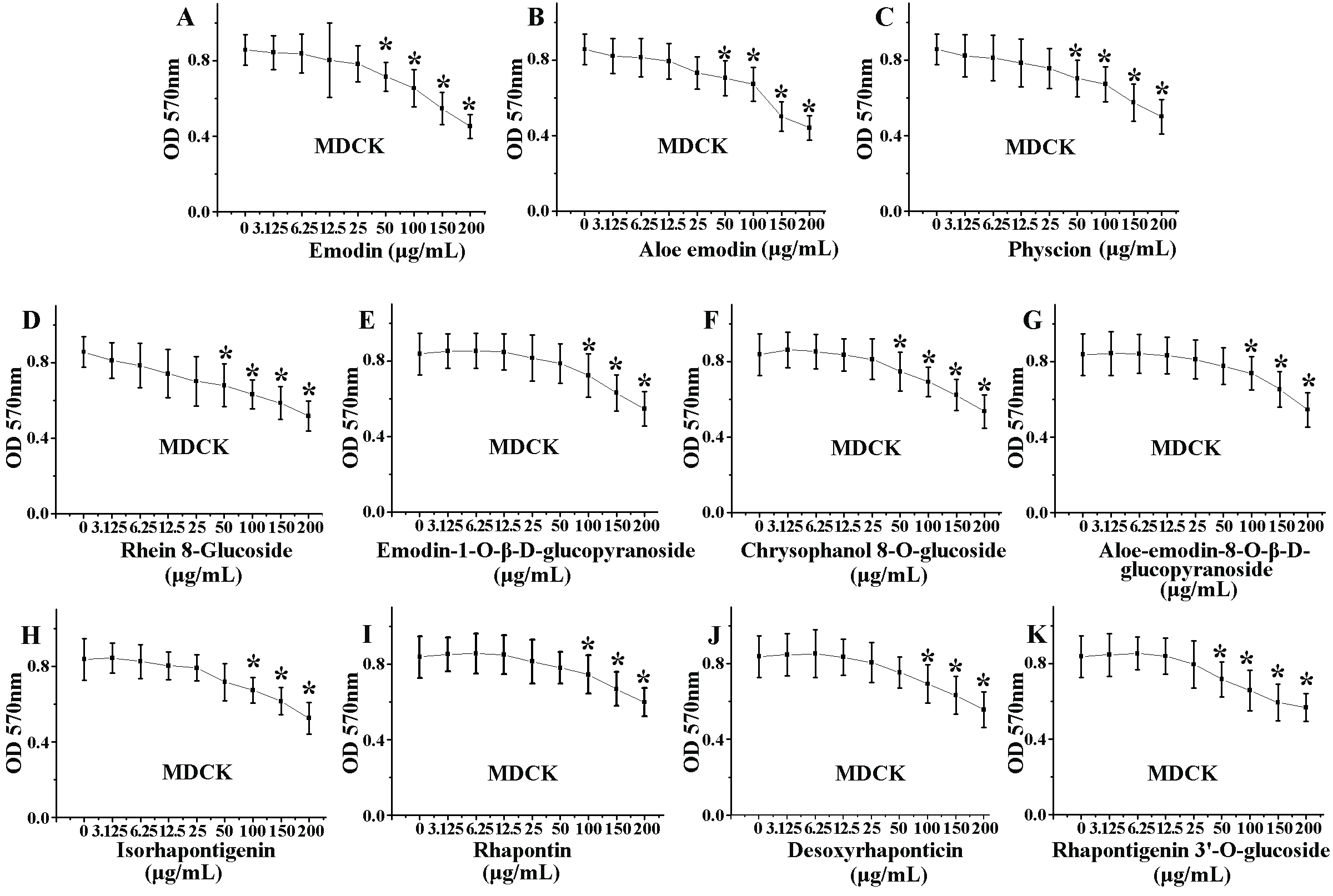


**Supplement figure 3.** The cytotoxicity of emodin and its analogs on MDCK cells was determined by a MTT assay. Data were the mean ± SD of three independent experiments each performed in triplicate. * *P* < 0.05 *vs.* the 0 µg/mL control.


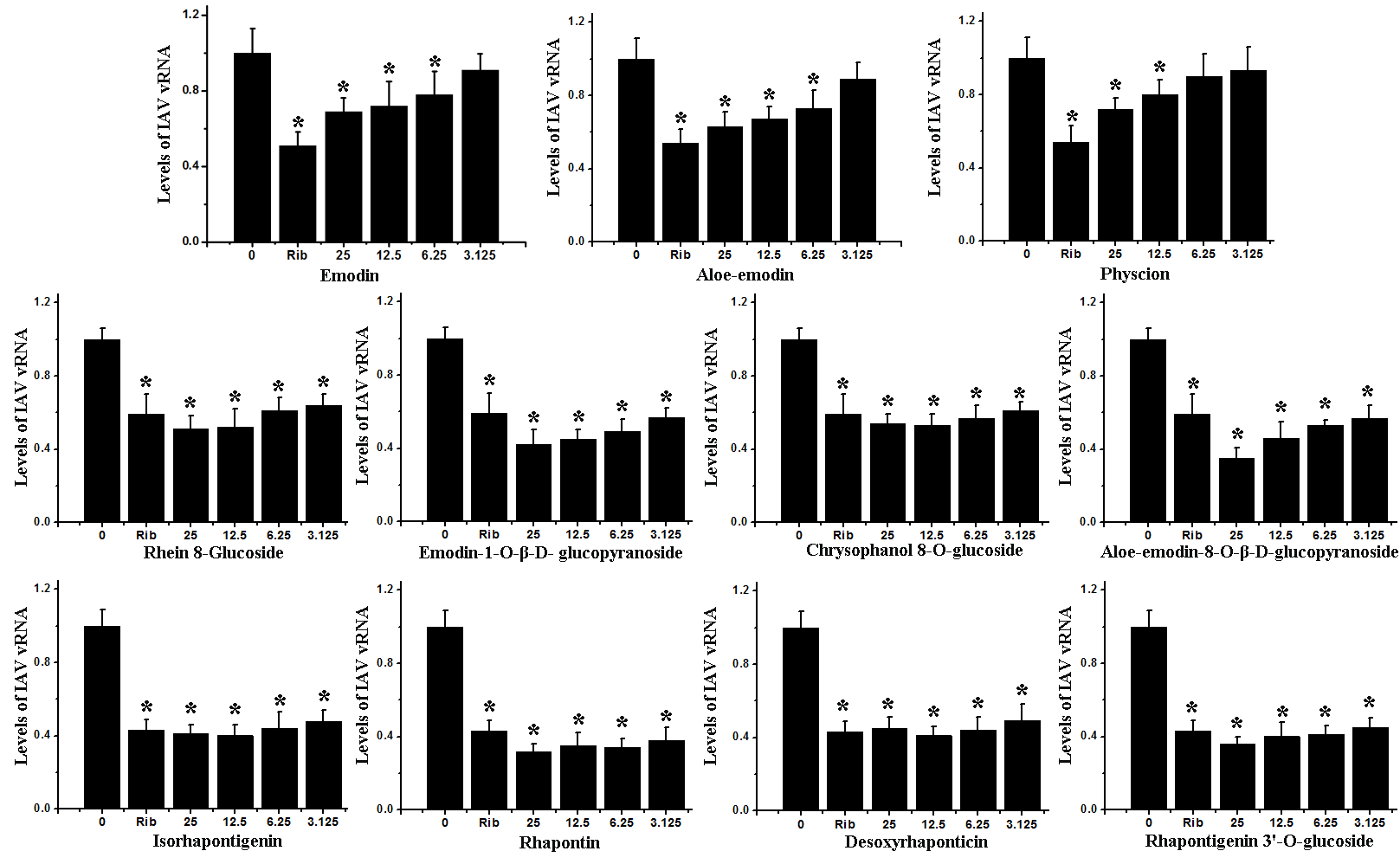


**Supplement figure 4.** The anti-IAV activity of emodin and its analogs was detected by a qRT-PCR assay. After infected with IAV (PR8, MOI = 0.001), A549 cells were treated with DMSO (< 0.5%), ribavirin (25 μg/mL), emodin or its analogs (25, 12.5, 6.25, and 3.125 μg/mL), respectively. MOI = 0.001. The incubation time was 48 h. All data shown were the mean ± SD of three independent experiments each performed in triplicate. **P* < 0.05 *vs*. 0 μg/mL group.


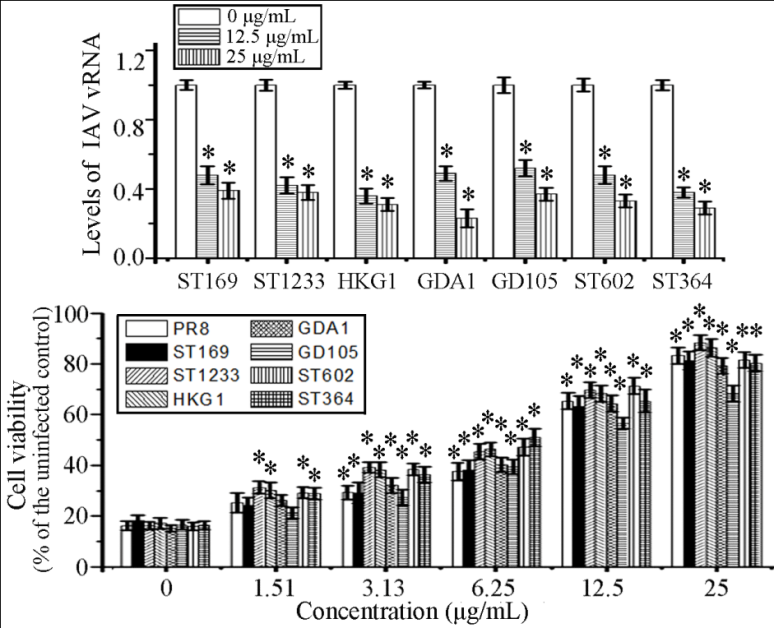


**Supplement figure 5**. The broad-spectrum antiviral activity of emodin *in vitro*. The broad-spectrum anti-IAV activity of emodin was detected using eight IAV strains, including PR8 (H1N1), ST169 (H1N1), ST1233 (H1N1), HKG1 (H9N2), GDA1 (H9N2), GD105 (H5N1), ST602 (H3N2), and ST364 (H3N2), by a qRT-PCR assay and a MTT assay. MOI = 0.001. The incubation time was 48 h. All data shown were the mean ± SD of three independent experiments each performed in triplicate. **P* < 0.05 *vs*. 0 μg/mL group.


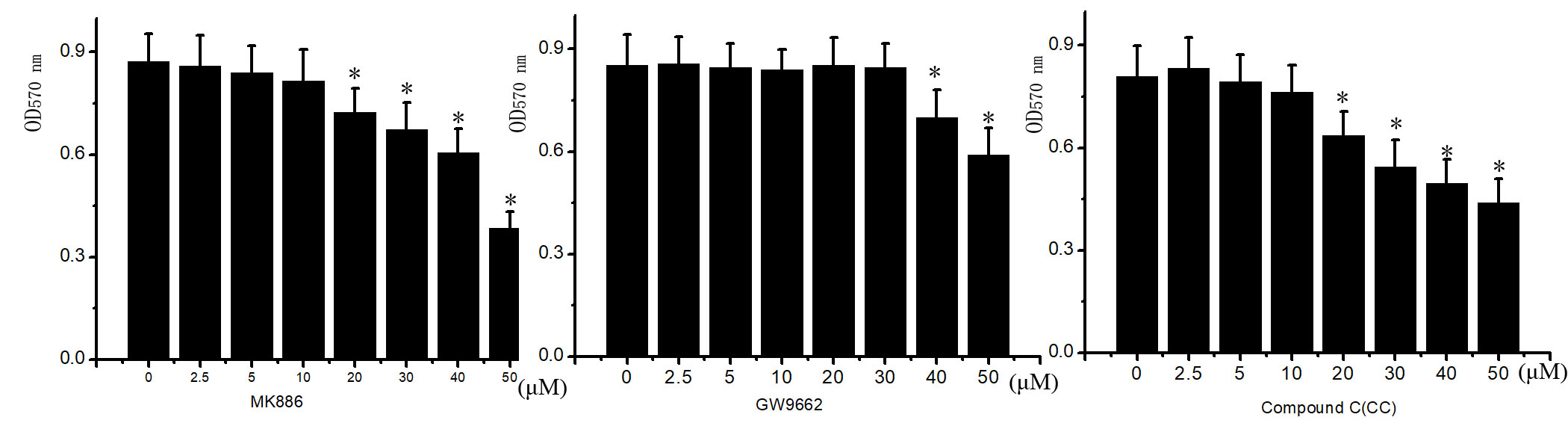


**Supplement figure 6**.The cytotoxicity of these inhibitors were determined at 48 h by a MTT assay. Data were the mean ± SD of three independent experiments each performed in triplicate. * *P* < 0.05 *vs.* the 0 µg/mL control.
